# Weekly Crime Concentration

**DOI:** 10.1007/s10940-021-09533-6

**Published:** 2021-09-01

**Authors:** Rafael Prieto Curiel

**Affiliations:** Centre for Advanced Spatial Analysis, University College London, London, UK

**Keywords:** Concentration of crime, Time series, Heartbeat

## Abstract

**Objectives:**

Examine and visualise the temporal concentration of different crime types and detect if their intensity varies through distinct moments of the week.

**Methods:**

The “heartbeat of the crime signal” is constructed by overlapping the weekly time they were suffered. This study is based on more than 220,000 crimes reported to the Mexico City Police Department between January 2016 and March 2020 to capture the day and time of crimes and detect moments of the week in which the intensity exceeds the average frequency. A new metric for the temporal concentration of crime is constructed for different types of crime and regions of the city based on the corresponding heartbeats.

**Results:**

The temporal concentration of crime is a stable signature of different types of crime. The intensity of robberies and theft is more homogeneous from Monday to Sunday, but robberies of a bank user are highly concentrated in a week, meaning that few hours of the week capture most of the burning moments. The concentration is not homogeneously distributed in the city, with some regions experiencing a much higher temporal concentration of crime.

**Conclusions:**

Crime is highly concentrated when observed in its weekly patterns, but different types of crime and regions exhibit substantially distinct concentration levels. The temporal trace indicates specific moments for the burning times of different types of crime, which is a critical element of a policing strategy.

## Introduction

Crime is concentrated in a few places, a reduced number of victims suffers it, and a small number of perpetrators commits it. This general pattern is observed, regardless of the location being analysed, the study’s period or the type of crime considered (Johnson 2010). There are several aspects in which its concentration is analysed, including the *victims* who suffer the crimes (SooHyun et al. 2017), the *offenders* who commit the crimes (Martinez et al. 2017; Farrington et al. 2001), and the *places* in which crime is executed (Weisburd 2015; Freeman 1996; Lee et al. 2017; Oliveira et al. 2017). Although far from being a perfect description, such as a physical law, the *Law of Crime Concentration* (Weisburd 2015) provides a general framework to consider the distinct aspects of crime in which it aggregates in non-random patterns. The concentration has been validated under a variety of scenarios (Hardyns et al. 2019; Favarin 2018; Amemiya and Ohyama 2019; Gill et al. 2017) including, for instance, street segments in some Latin American cities (Jaitman and Ajzenman 2016), breaking-and-entering at the household, street segment, and neighbourhood levels in a city in Nigeria (Umar et al. 2021), terrorism in Jerusalem (Perry 2020), and opioid overdose deaths (Carter et al. 2019) among many more.

The fact that crime is concentrated in space results in hotspots of crime, which has many implications in terms of policing and crime prevention, since it means that the allocation of resources can be done efficiently, targeting with a higher emphasis the victims, places or potential criminals who are more likely to suffer (or commit) a crime (Levin et al. 2017). Crime concentration is a key element for designing and measuring crime reduction strategies (Rogerson 2008), accounting for the public health burden (Carter et al. 2019) and detecting the impacts of crime on the real estate market (Ceccato and Wilhelmsson 2020) among many. The fact that crime concentrates among some individuals or is perpetrated only by a limited number of people also impacts prevention programs and is a crucial driver of fear of crime (Prieto Curiel and Bishop 2017).

Crime and other social phenomena are human activities performed over space and time (Cohen and Felson 1979). The temporal concentration of crime is also a major part of crime analysis. The intensity of crime varies seasonally, weekly, daily, and hourly with specific *burning* (high intensity) and *freezing* (low intensity) times (Brantingham et al. 2020). As relevant as other dimensions of crime concentration, the temporal trace of crime is critical to detect the factors that structure criminal opportunities. Time, however, is much more ambiguous than observing the victims, the offenders, or the locations. When observed as the progression of moments, data exhibits trends and shocks, such as the crime drop in some parts of the World (Pease and Ignatans 2016). Yet, time is more flexible and can be analysed with other techniques by matching distinct moments as the same unit of observation. For instance, when distinct moments of a year are paired (so February is matched with other Februarys, even if they are years apart), it is possible to detect annual variations (Oliveira et al. 2018). With a yearly analysis, we can detect seasonality in the data and observe that fluctuations have environmental and social components, which can combine to create different patterns from one location to another (Phelps 1929; McDowall et al. 2012; Andresen and Malleson 2013). When Fridays are matched with Fridays, weekly patterns are observed, but when 18:00 is paired with other 18:00s, daily cycles are observed, making it possible to detect hour-to-hour variations (Bernasco et al. 2017) or to detect cycles in crime incidence (Van Koppen and Jansen 1999; Valente 2019; Brunsdon and Corcoran 2006). Yet, there are many more ways to match distinct moments in time with others, for instance, school days and non-school days (Bernasco et al. 2017), weekdays and weekends (Brunsdon and Corcoran 2006), economic cycles (Cook and Zarkin 1985), and other temporal units (Cohn and Breetzke 2017).

Significant advances in how to analyse and what to expect concerning the concentration of crime in places and among victims or offenders have not been matched with analytical tools to investigate crime’s temporal concentration. When time is observed as the progression of consecutive moments, crime analysis is often based on time-series techniques, such as auto-regressive models or even Fourier analysis (Cohn and Breetzke 2017). Yet, the most relevant temporal unit to analyse social events are the weeks. Weekly cycles are the patterns formed by most social events, such as the number of customers in a shop or a bar or the number of students and teachers in a school. Weekly cycles, which emerge as a result of weekly commuting patterns of work, education and leisure, are also observed in temporal crime patterns (Prieto Curiel et al. 2021). However, in terms of crime, weekly cycles are often unexplored. Whether a few hours of the week concentrate most of the crimes and whether the observed temporal patterns hold for different parts of the city is still unknown. The range of variation of the intensity of crimes between the burning and the freezing moments of the week and whether it is similar for distinct types of crime is unknown but has an impact as significant as the concentration of crime among victims, places and offenders. A considerable variation of the intensity between the burning and the freezing moments should be a central element for planning and forecasting the demand for security resources or emergency calls, planning dispatcher shifts, and policing. Furthermore, are those weekly and daily cycles stable across time, or is their variation so large that it makes it almost impossible to use previous events’ temporal trace to forecast future weekly and daily cycles? How similar is, in terms of crime, one week after the next one? And  one day after the next one? How many minutes or hours are needed to concentrate most of the crimes on a day or a week?

Here, daily and weekly crime cycles are explored, following a technique to detect moments with a higher and lower intensity of events (Prieto Curiel et al. 2021), or “heartbeat of the crime signal”, or simply the “heartbeat”. The technique results in a visualisation of the crime cycles so that it is easy to detect moments of the day and the week with higher and lower intensities of crime. Using data related to violent crimes from Mexico City gives the time of more than 220,000 crimes between January 2016 and March 2020 (before the COVID-19 pandemic), and the heartbeats of different types of crime are explored. A new metric for the temporal concentration of crime is constructed. Results show that the temporal trace of crimes is highly concentrated and stable across the years, far from what randomness would yield, but is also substantially different among distinct types of crime. The occurrence of some activity-specific types of crime, such as robberies of a bank user or crimes suffered by food deliveries have the highest temporal concentration, with 72% and 56% of the total intensity of crime occurring in the top 20% of the time of the week, whereas in crimes which do not rely on specific activities, such as robbery of a person, 32% of the total intensity of crime happens in the top 20% most dangerous moments of the week.

## Crime is a Temporal Event

According to *Routine Activity Theory*, a crime occurs when a motivated offender, a suitable target, and the right conditions for such crime (for example, the absence of guardians or the excess of people for the criminal to go unadvertised) converge in space and in time (Cohen and Felson 1979), in settings that make committing the crime easy, safe and profitable (Brantingham and Brantingham 2010). But the “right conditions” for such crime are most likely identical the day after and the week after. The busy street, the dark alley or the crowded market will be just as busy, dark or crowded in similar times of the day and days of the week, so the conditions for similar crimes to happen in the future keep converging. Everyday life, characterised by daily and weekly routines, explains why crime occurs in recurrent spaces and times.

Some environmental factors which work as attractors or as generators of crime function in a time-dependant manner (Brantingham and Brantingham 2010). A shop, a bar or a school attract customers, teachers, and students at certain moments of the week and thus, alter the opportunities of a crime by making it more or less profitable and easier or harder to be executed. The impact, however, should be observed close to when bars are operating, shops are open, or schools are functioning, and so cycles in terms of when crime happens should be observed (Prieto Curiel et al. 2021). Commuters alter the population’s distribution substantially in a city (Andresen 2010), and therefore, can have a major impact on crime rates (Felson and Boivin 2015; Stults and Hasbrouck 2015). Neighbourhoods fluctuate as a result of human mobility but also as a result of the activities that take place in that neighbourhood, altering the intersection in space and time of potential offenders, victims, and guardians (Browning et al. 2021).

The temporal dimension has been a central part of crime analysis. For example, yearly rates in some US states were used to detect crime cycles nearly a century ago (Phelps 1929). Since then, many aspects of the temporal signature of crimes have been observed, including yearly trends (Townsley 2008), seasonality (Hird and Ruparel 2007; McDowall et al. 2012; Andresen and Malleson 2013) and other temporal signals (Cohn and Breetzke 2017; Oliveira et al. 2018). The spatio-temporal dimension has also been analysed for optimal policing (Mukhopadhyay et al. 2016) or to study demographic covariates and their impact on violence interruption (Park et al. 2019). Further, it has been observed that spatio-temporal patterns vary according to different types of crime (de Melo et al. 2018) and that hotspots change across space depending on the time of the day (Valente 2019). The temporal dimension of crime plays a central role in the understanding of crime patterns. It was suggested that yearly trends and a schedule with burning and freezing crime moments should be a standard part of hotspot maps to analyse crime patterns (Townsley 2008).

### Weekly Cycles of Crime

By dividing the 24 h of the day into four non-overlapping periods of different duration, it was observed that potentially criminogenic facilities, including ATMs, alcohol stores, bars, fast-food restaurants, pawnshops, and others, might facilitate street robbery opportunities when open and in use. Still, some places may also facilitate street robbery opportunities during other times as well (Haberman and Ratcliffe 2015). Also, by considering 2-h time blocks for each day of the week, it was detected in a city in the US that street robbers prefer to attack near cash-intensive businesses even when they are closed and therefore with low activity levels (Bernasco et al. 2017).

One of the common problems when dealing with temporal data is the use of arbitrary grids, which depend on two (or more) parameters: the length of the cells and their starting point. Considering 1-h or 2-h cells could alter results as much as considering slots from 14:00 to 14:59 or from 14:01 to 15:00, particularly in the case of crime, where data is often obtained by the victims’ reports, and there is a very high tendency of reporting events at even hours (Prieto Curiel et al. 2021). Furthermore, the use of arbitrary grids is often quite challenging when data has a relatively low frequency. With low-frequency data, such as crime, there will always be crime-free regions in space (Levin et al. 2017; Bernasco and Steenbeek 2017) and the same occurs in time, where for a sufficiently refined grid (a partition with short periods of time), most observations are zero, and the counts become a rare event. Often, large periods are used to overcome the low frequency of crime, for instance, up to nearly 10 h (Haberman and Ratcliffe 2015), which in turn has the cost of assuming that the observed conditions at 22:00 and 6:30 the day after are similar (or at least similar enough not to divide them into more units) and possibly the cost of comparing long and short intervals of time.

Perhaps the highest cost of using arbitrary grids for the temporal analysis of crime is the trade-off between having a very limited number of observations (maybe four, eight or twelve observations per day), which does not provide enough data points to correlate moments or other types of analysis, or dividing time into more intervals, but then having most counts with zero (or close to zero) events.

An appropriate technique to overcome grids is to “smooth” the data using a one-dimensional kernel density estimate. The technique is frequently used for two-dimensional data in crime science to produce hotspot maps, which reduces the dependence on arbitrary parameters. An additive model attempts to quantify the background intensity, or rate, by smoothing the observed moments in which events happened and has been used to study temporal signatures of crime (Park et al. 2019; Brunsdon and Corcoran 2006; Prieto Curiel et al. 2021). A kernel density estimate does depend on a smoothing-function selection (often Gaussian, but others, such as a double exponential could be used) and a parameter, the bandwidth, which is the range in which a crime has an impact on the computation of the background intensity. And, although no perfect technique for choosing smoothing function or bandwidth exists [such as Silverman’s rule (Silverman 1986)], a visual inspection of the smoothed data is often enough to detect if the bandwidth picked is too large (so the smoothed data becomes too flat) or too small (so the smoothed data varies too fast). Based on a kernel density estimate for weekly data, the use of circular statistics was proposed to capture the convergence of potential victims, motivated offenders, and the right conditions (Brunsdon and Corcoran 2006). The use of circular statistics in crime science allowed modelling daily cycles and their visualisation, also applying a time-in-week analysis for reported incidents in a city in Wales (Brunsdon and Corcoran 2006).

Still, with smoothed data and circular statistics, the level of temporal concentration of crime remains unexplored. A novel technique based on a Gaussian additive kernel density estimate was proposed to analyse weekly cycles of crime and crashes data, named “heartbeats” since the resulting curve resembles an electrocardiogram (Prieto Curiel et al. 2021). The technique differentiates weekdays from weekends, Mondays from Tuesdays and mornings from evenings and nights in a continuous manner by smoothing the moment in which crimes happened. The heartbeats are used here to measure the temporal concentration of crime, detect the variation observed through a week for different types of crime, and check whether the observed temporal concentration is stable across space and throughout the years.

## Methods

Weekly crime heartbeats are constructed by smoothing the date in which a crime happened (Prieto Curiel et al. 2021). To consider time cyclically, the time of the week in which a crime happened is considered. Each crime is represented by its moment of the week, $t_i \in [0, 7)$, where, for example, $t_i = 0.75$ means that the $i$th crime happened on a Monday at 18:00 and $t_i= 6.96$ means that it happened on a Sunday at 23:00. The moment in which crimes occur is a point process (or a discrete event) in time, but the risk is not. The rate (or risk) of a crime happening at a certain moment of the week is estimated by smoothing the data using an additive Gaussian kernel density estimate (Mohler et al. 2011; Park et al. 2019). The heartbeat of the crime signal, *H*(*t*), is defined by

1 $$\begin{aligned} H(t) = \sum _{i} \exp \left( -\frac{(t-s_i)^2}{2\omega ^2} \right) , \end{aligned}$$

where $s_i = \mathrm{arg}\,\mathrm{min}\left( |t-t_i-7|, |t-t_i|, |t-t_i+7|\right)$ gives the smooth data its cyclic behaviour; $\omega$ is the bandwidth of the smoothing process and the sum of the kernels is computed over all the crimes. The bandwidth $\omega$ is chosen such that for a single crime with time $t_i$, its heartbeat $H(t_i)=1$ and $H(t_i \pm 2/24) = 0.2$, meaning that 2 h after (or before) the crime, the smooth rate decrease to 0.2. The function *H*(*t*) should, in general, resemble an electrocardiogram, with daily and weekly cycles and maybe with specific peaks around rush hour, lunchtime or other events which tend to synchronise social activities. The smoothing helps to ignore the propensity of crimes to be reported at even hours or 15-min breaks. See the “Mathematical Formality” of the Appendix for more details about the mathematical construction of the heartbeats.

The temporal concentration of crime, $\chi$, is defined as the Gini index of $H(t)$. A value of $\chi$ close to zero means that crime occurs with a similar intensity during the week, and values of $\chi$ close to one represent that crime is concentrated at specific moments of the week. The concentration can be compared between different types of crime since the Gini index does not depend on the scale, so that the concentration $\chi$ depends only on the weekly patterns of crime and not its volume.

The heartbeat $H(t)$ does depend on the number of crimes considered. For comparing between different types of crime, or periods in which the volume of crime might vary, the weekly variation $\eta (t)$ is constructed by comparing the heartbeat against its mean, such that

2 $$\begin{aligned} \eta (t) =\frac{H(t)-\mu }{\mu }, \end{aligned}$$

where $\mu$ is the average intensity of $H(t)$. The average intensity $\mu$ only depends on the number of crimes and not on their exact date, meaning that its value is the same for all heartbeats with the same number of crimes, even if their shape is different. The minimum value of the weekly variation $\eta (t)$ is $-1$ when the rate is zero at time *t*. When $\eta (t)=0$, the intensity at time *t* is equal to the average. With $\eta (t)=1$, the intensity of crime is double the average, and so on. The values $\eta (t)$ can be compared between distinct types of crime, even if the number of crimes is different.

The heartbeat is not invariant to the amount of data, so in general, with more observations (that is, more crime), $H(t)$ will have larger values. However, for a sufficiently large dataset, adding more observations with the same temporal structure does not affect the values of the weekly variation $\eta (t)$ and does not modify the concentration metric $\chi$ either. That means that $\eta (t)$ and the temporal concentration metric $\chi$ are invariant to the amount of data considered. In turn, it is possible to compare both $\eta (t)$ and $\chi$ for types of crime with different volumes and any discrepancies are due to a different temporal signature.

### Is the Heartbeat the Result of Randomness?

**Fig. 1:**
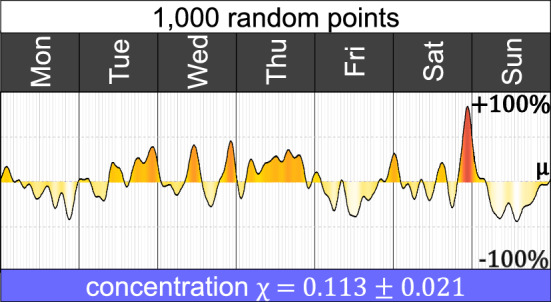
Heartbeat of 1000 randomly distributed observations in the [0, 7) interval. Intervals are obtained by repeating 500 times the simulation process and dropping the extreme values

Are the heartbeat $H(t)$, the concentration metric $\chi$ and the weekly variation $\eta (t)$ the result of randomness? All temporal events, particularly if they are scarce, will show some pattern and exhibit a temporal concentration, but it is relevant to check if that perceived pattern is only the result of randomness. Therefore, detecting how a heartbeat and a concentration metric of randomly distributed data are valuable starting points (Chalfin et al. 2021). Consider $N = 1000$ randomly-distributed points in the [0, 7) interval and construct its corresponding heartbeat $H_N(t)$, its weekly variation $\eta _N(t)$ and its concentration $\chi _N$. With $N = 1000$ random points, it is easy to detect how a random temporal pattern looks (Fig. 1).

With $N = 1000$ random points, some peaks might be observed, but they do not form daily cyclic patterns. The heartbeat produced with random data fluctuates rapidly around its mean. The concentration metric gives $\chi _N = 0.113 \pm 0.021$. A null hypothesis of randomness can be constructed by comparing the observed concentration metric $\chi$ for some data against the simulated metric $\chi _N$. There are two reasons why the null hypothesis could be rejected. Firstly, if the observed concentration is above the simulated interval. Such a scenario means that the data has some temporal (non-random) structure, with some moments of the week hotter and other moments colder than the weekly mean. Secondly, suppose the observed concentration is below the simulated interval. In that case, randomness is also rejected, but it means that the data is more uniformly distributed than randomness (for example, with one event every 10 min).

A similar test can be constructed with different volumes of data, where simulated data helps obtain random heartbeats $H_N(t)$ and intervals $\chi _N$ for rejecting a null hypothesis of randomness.

### Data

Open-access data from Mexico City, from January 2016 to March 2020 (pre-pandemic) available at https://datos.cdmx.gob.mx/ gives the time and location of more than one million crimes reported to the Investigative Police. For some crimes in which there is no present victim (for example, vehicle theft without violence), it is not always possible to identify the precise moment in which it happened. In contrast, for other types of crime (for example, fraud or extortion), the actual moment or location of the crime is not clear. To keep the precision in terms of the crime’s time and location, only robberies with a present victim are kept. In total, 222,741 crimes are analysed (21.8% of the reported data). Considering the minute when a crime happened, roughly $1/60 = 1.6\%$ should be reported at even hours (such as 19:00) but nearly 33% are, plus 25% reported on half-hour intervals (such as 21:30) so the variable is biased towards specific moments of the day. See the “Considering only Property Crimes with a Present Victim” of the Appendix for more details about the selection process and some statistics about the crimes considered.

According to the Mexican Victimisation Survey ENVIPE, in Mexico City, only 6% of crimes are reported (INEGI 2019). This is problematic from many angles as the data is biased. However, the time in which the crime happened should not significantly alter whether it gets reported to the police or not. Thus, the shape of the heartbeat should not be altered much by the number of underreported crimes and the same for the weekly variation $\eta (t)$ and the concentration metric since both are volume-invariant. The heartbeat of the crime signal for the whole city is constructed using more than 220,000 crimes which is more than enough for detecting the weekly trace. In fact, if instead of 6% of the crimes, only 1% of the crimes were reported to the police, the heartbeat, the weekly variation, and the concentration metric would be roughly the same (provided that the 1% and the 6% that gets reported is randomly selected from all crimes). See the “Under-Reported Crime” of the Appendix for the construction of heartbeats based on a smaller percentage of crimes.

The heartbeat for all the city, for all robberies and for all the time considered is valuable, but it is more informative to construct the heartbeat by years, by type of crime or by season and detect any structural differences. Seven types of crime are considered (with their corresponding frequencies in Table 1). The most frequent type of crime is robbery of a person, with nearly 100,000 reports, and the least frequent one is robbery of a bank user (frequently committed right after a person used an ATM or went inside a bank) with fewer than 2000 crimes.

**Table 1:** Frequency of some types of crimes reported to the Police in Mexico City between January 2016 and March 2020

Type of crime	Frequency	%
Robbery of a person	96,711	43.4
Theft	60,024	26.9
Victim in public transport	20,839	9.4
Carjacking	17,666	7.9
Business	15,838	7.1
Delivery person	9.780	4.3
Robbery of a bank user	1883	0.8
Total	222,741	100

Only property crimes with a present victim are considered here

There are 1551 days between January 2016 and March 2020, meaning a daily rate of 1.2 robberies of bank users. With little more than one crime per day, moments of the week with a higher intensity can only be detected when cyclic time is considered. Still, for more frequent types of crime, observing 6.3 daily reports, as in crimes suffered by people who deliver, or 10.2 daily reports of robbery of a business and detecting the daily or weekly trace is impossible if weekly cycles are ignored.

For different types of crime, its corresponding heartbeat $H_R(t)$, concentration $\chi _R$ and weekly variation $\eta _R(t)$ are constructed. Also, using the coordinates in which the crime is reported, it is possible to compute the heartbeat of different regions of the city by considering only crimes inside that region.

## Results

**Fig. 2:**
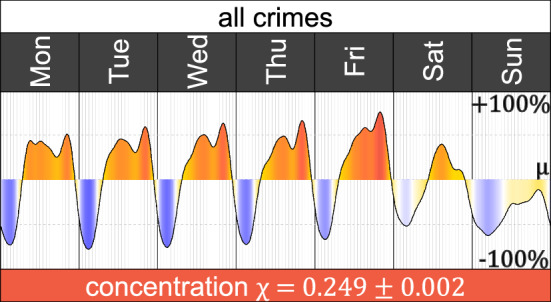
Weekly variation of the heartbeat of crime $\eta (t)$ which shows when the intensity is above or below the observed average of the week, $\mu$. When the intensity is below the mean $\mu$, the polygon is dark blue; when it is around the mean, the polygon is light yellow, and when the intensity is above the mean, the polygon is red (Color figure online)

Crimes form a daily and a weekly pattern, dominated mainly through our daily activities, routines, commutes, and land use (Prieto Curiel et al. 2021). The weekly variation of the heartbeat, $\eta (t)$, reveals that weekdays are similar. They shift from a low intensity of crime in the early hours of the day and the morning to a high intensity, maintained from around 10:00 to 22:00. From Monday to Friday, there are two peaks with high intensity. The first is the early morning one, and the second is during the evening, more pronounced on Fridays. Saturdays have a different pattern, with a higher intensity of crime than other days during the first hours of the day, to a single peak in the afternoon. Sundays remain below the average, $\mu$, during the whole day, although late Sunday, the heartbeat approaches values closer to the mean (Fig. 2).

The concentration of crime gives $\chi = 0.249 \pm 0.002$, which is far from the 95% randomness interval (0.006, 0.009) for such volume of crimes and therefore, the observed concentration is not obtained simply by chance. The heartbeat shows a non-random pattern with a much higher intensity of crimes at certain times, resulting from activities dominated by weekly cycles such as commutes and leisure activities.

The weekly variation of the heartbeat, $\eta (t)$, ranges from $-\,77\%$ on early hours of a Tuesday to $+\,75\%$ on Friday evening, which means that if there are $\mu = 100$ crimes per hour on average, the city shifts from 23 crimes per hour to 175 crimes, so there are 7.6 times more crime during a burning hour than a freezing hour.

### Heartbeats by Type of Crime

**Fig. 3:**
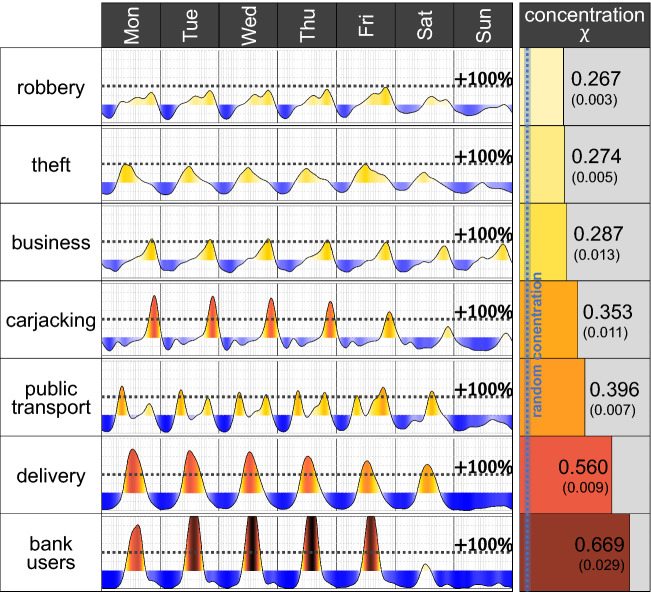
Heartbeat of different types of crime $\eta _R(t)$ which shows moments of the week when the intensity of that type of crime is above or below the observed average $\mu _R$ (left). Concentration metric $\chi _R$ for different types of crime including a $95\%$ interval and concentration for a randomly constructed heartbeat

The weekly variation of the heartbeat of the signal of different types of crime shows that weekly patterns are substantially different. The heartbeat of robberies $H_R(t)$ and of theft $H_T(t)$, for example, show that, in general, nights have a lower intensity than the days. However, the highest intensity of theft is reached before midday, while for robberies, the maximum intensity is observed much later. In the case of robberies, for instance, during late Fridays, the heartbeat reaches its maximum peak and doubles the average intensity observed in the rest of the week (Fig. 3).

In the case of robbery of a business, it usually doubles its average intensity around 21:00. Interestingly, weekdays and weekends are roughly the same and follow similar daily patterns, unlike other types of crime, where the weekend is substantially less active.

Carjacking is a crime that happens mostly between 21:00 and 23:00 from Monday to Friday, although Friday has a smaller peak. During the burning times of carjacking, the intensity more than triples the average intensity observed during the rest of the week and is 13 times higher than the intensity during the freezing moments.

Crimes against public transport passengers have two daily peaks from Monday to Friday, synchronising with commuters’ rush hour. However, on Saturday, there is only one peak in the afternoon and after, the crime has a very low intensity, roughly until Monday morning.

Crimes suffered by people who deliver food and other goods to shops and houses have a pattern with daily peaks during midday and reaches an intensity close to zero around midnight from Monday to Saturday. There are nearly zero crimes on Sundays. The highest intensity of the weekly variation $\eta_D(t)$ reaches $+\,250\%$, meaning that it is 3.5 times the average intensity observed during the week. Also, the highest daily peak of $\eta _D(t)$ is roughly the same from Monday to Wednesday, but it is slightly less pronounced as the week goes by, and the peak on Friday is $+\,170\%$ and on Saturday is $+\,155\%$. Thus, the heartbeat of the crimes suffered by people who deliver food and other goods is matched by moments in which most shop deliveries are made.

Robberies of a bank user also have a daily peak from Monday to Friday and a smaller peak on Saturdays. The highest intensity of $\eta _B(t)$ is $+\,411\%$, meaning that on Thursdays, the intensity is more than five times the average intensity of the week. From the peak observed on Saturday, there are nearly no crimes on Sunday and $\eta _B(t)=-100\%$.

The temporal trace observed across different types of crimes is substantially distinct, with peaks at different days and times of the week and increases and decreases in their intensity. The observed city-level heartbeat (Fig. 2) is, in essence, a combination of the crime-specific heartbeats (Fig. 3), weighted by the volume of each type of crime.

### Temporal Concentration of Crime

The levels of temporal concentration for different types of crime varies substantially from $\chi _R = 0.267$ for robbery of a person, to $\chi _B = 0.669$ for robbery of a bank user (right panel of Fig. 3). None of the observed levels of concentration is the result of randomness.

For crimes against bank users and against people who deliver goods, the temporal concentration is surprisingly high. The top 10% most criminal moments of the week (144 min or roughly 2.5 h per day) concentrate 44% of the robberies of a bank user and 31% of the crimes against people who deliver goods. The least-concentrated crimes are robbery of a person and theft, where the top 10% most intense moments concentrate 17% and 18% of the crime intensity, respectively. Allocating resources, planning a security program or designing a prevention strategy against a specific type of crime should be substantially different if in less than 3 h per day occur more than half of the crimes, as in the case of robbery of a bank user, or less than one-fifth, as in the case of robbery of a person.

### Temporal Correlation by Type of Crime

**Fig. 4:**
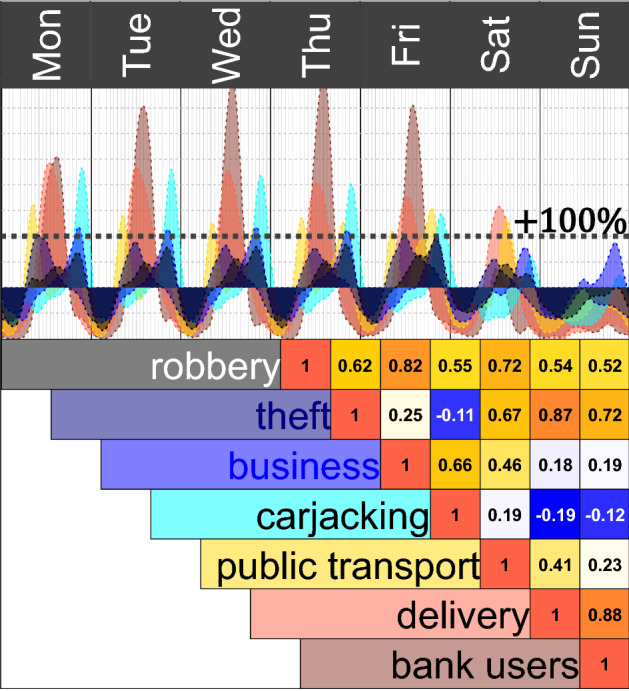
Heartbeat per type of crime (top) and their pairwise correlation (bottom) where a higher level of correlation is coloured by a darker red shade

Different types of crime have a distinct heartbeat. For example, during some moments of the week, there is a high intensity of crimes against public transport users, and at different moments, there is a high intensity of carjacking or thefts. The correlation between the heartbeats per type of crime show that some might co-occur during the week, for example, crimes suffered by people who deliver food and other goods and suffered by bank users, with a correlation $cor(\eta _D(t), \eta _B(t)) = 0.88$ (Fig. 4). However, the correlation is negative for some types of crime, including carjacking and theft, or carjacking and robberies of a bank user, meaning that those types of crime have little temporal co-occurrence. All types of crime have, in general, a low intensity during the first hours of each day and high intensity at some point during the day, although the precise moments and the daily shifts can be substantially different.

Between two random (simulated) heartbeats, a 95% interval of the observed correlation is $(-\,0.26, 0.26)$, meaning that the observed correlation between theft and carjacking, for instance, or between crimes suffered by people who deliver and carjacking could be observed by randomness. The observed pairwise correlation of every other two types of crime is positive and outside the randomness interval.

### Heartbeats Across Different Regions

According to many local factors, including the land use, the heartbeats change across different regions of the city (Prieto Curiel et al. 2021). However, those changes could result from an homogeneous heartbeat per type of crime combined with different frequencies in distinct parts of the city. For example, if some neighbourhood has a higher frequency of robberies of a bank user, its heartbeat would be different from a neighbourhood with many carjackings, simply because of the different composition of crimes. However, this is not the case. Heartbeats are not necessarily homogeneous across the city. Dividing the city area into four concentric circles centred in the city’s main square (Zócalo) helps reject this hypothesis.

Formally, crimes inside a disc centred in the main square with a 2.5 km radius are considered to construct the heartbeat of the central part of the city. This part can be regarded as the city centre, with many government agencies and commercial districts, so it attracts people from other parts of the metropolitan area. Crimes that occurred inside the two non-overlapping concentric rings, with an extra 2.5 km each, are then used to construct the heartbeat of the consecutive central regions of Mexico City. These two regions are still close enough to the city centre and tend to be more residential than the city centre. Finally, crimes at a distance larger than 7.5 km are considered to construct the corresponding heartbeats of the peripheral parts of the city. With this partition, all crimes are considered in any of the four regions, so it allows comparing the heartbeat of the city centre, two outer rings and the peripheral parts of the city. To build the four heartbeats, only the most frequent type of crime, robbery of a person, is considered so that any observed difference in the heartbeats is only due to a different temporal pattern and not the composition of crimes. Although classifying crimes according to their distance to the city centre into four regions is arbitrary, due to the higher volume of robberies near the city centre, gives four regions with a similar amount of crimes, from 13 thousand in the central disc, 15 and 20 thousand crimes in the two rings and 48 thousand in the peripheral part.

**Fig. 5:**
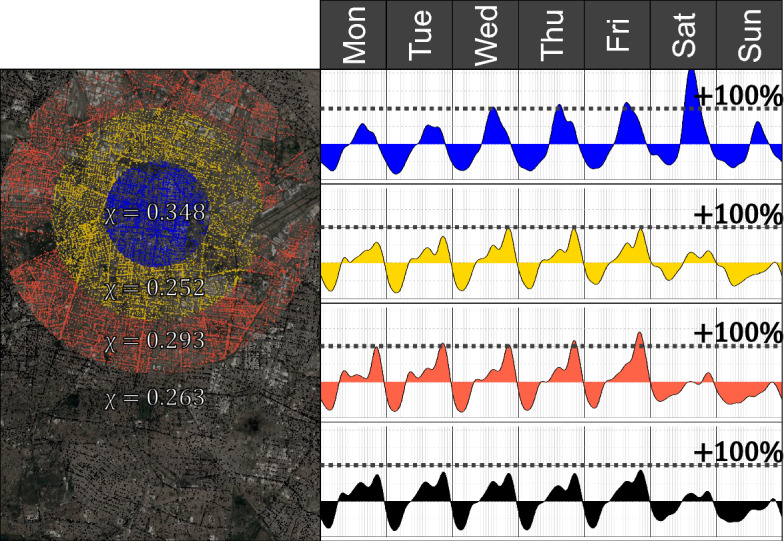
Heartbeat of crime $\eta_R(t)$ and the corresponding concentration $\chi _R$ for the most central part of the region (top, at a distance smaller than 2.5 km to city centre), two concentric rings (middle) and the outer part of the city (bottom, at a distance larger than 7.5 km to city centre). A different colour marks the location of each crime, depending on which area it happened (Color figure online)

Results show that the heartbeat of the signal of crimes in the central part of the city is substantially different to the other heartbeats, with a higher level of concentration $\chi _{R1} = 0.348$, compared to $\chi _{R2}= 0.252$ in the adjacent second ring (Fig. 5).

The heartbeat of robbery of a person in the city’s central part $H_{R1}(t)$ has a single peak from Monday to Saturday at around 16:00, and the intensity of that peak increases as the day passes by. On a Friday at 16:00, the intensity of crime is more than double the week’s average intensity, and on Saturday, it is more than three times the average intensity of the week. However, in the second ring (that is, the closest ring to the city centre), the highest intensity is only $\eta _{R2}=+100\%$ so that from Wednesday to Friday, the highest peak doubles the average intensity of the week. Also, the peak in that second ring is observed between 20:00 and 21:00, many hours after a peak is observed in the city centre. On a Saturday, there is the highest peak of the week in the city centre, but in the second ring, $\eta _{R2}(t)$ is just above the average intensity. On a Sunday, only the city’s central part goes above the average whilst the rest of the regions maintain a lower-than-average rate throughout the whole day. In the central parts of the city there are fewer crimes from 21:00 or 22:00 than the rest of the week, but in those hours, the outer rings are going through a daily peak and they go below zero some hours after the central region. A similar thing happens in the early hours of weekdays. At around 8:00, the outer rings generally observe an increase in crimes’ intensity, mainly from Monday to Wednesday. However, at those moments, the central part of the city is still below the average intensity and will increase a few hours later.

For a specific type of crime (robbery of a person in this case), the regional heartbeats show a substantially different temporal signal. In the most central part of the city, 23% of the crimes happen during the top 10% most crime moments of the week, but in its neighbouring ring, that is, the area around the city centre, only 17% of the crimes happen during its corresponding top 10% moments of the week. Furthermore, the top 10% moments of the week of the city’s central part (that is, detecting the 16.8 h of the week in which the central area has the highest intensity) overlap in less than 1 h with the top 10% moments of its neighbouring ring. Thus, even for the same type of crime, hotspots shift throughout the week from region to region and the burning moments in one region do not correspond with the burning moments of its neighbouring regions.

### Highest Intensity Moments of the Week

**Fig. 6:**
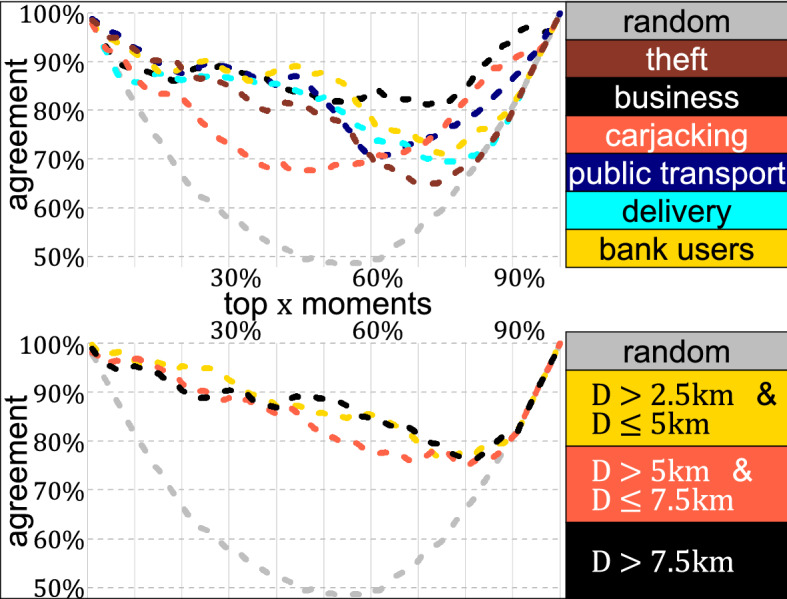
Agreement (vertical axis) of the binary classification of the top $x\%$ moments of the year (horizontal axis) between robbery of a person and other types of crime, and between robbery of a person and a randomly-constructed heartbeat (top part) and between the central part of the city and three concentric areas (bottom part). For $x=0\%$ and $x=100\%$, any two classifications are the same, but for other values, the level of disagreement shows how different are the heartbeats

Detecting the overlaps of the week’s most intense moments for different types of crime (and for different regions) reveals whether concentration patterns are similar and co-occur during the week. For each moment of the week, $t$, it is identified whether it belongs, or not, to the top $x\%$ most intense moments for a specific heartbeat *H*(*t*) or equivalently, the weekly variation $\eta _c(t)$. This process can be thought of as a binary classification process, $I_{\eta _c}(t, x)$, where $\eta _c(t)$ classifies whether *t* belongs in the top $x\%$ moments of the week (so $I_{\eta _c}(t, x)=1$) or not (so $I_{\eta _c}(t, x)=0$). With $x = 0$ the classification is $I_{\eta _c}(t, x)=0$ for all *t*, so no moment is classified as the top 0% and with $x = 100\%$ the classification is $I_{\eta _c}(t, x)=1$ so all moments are classified as part of the top 100% moments of the week. Based on the classification obtained, it is possible to compare the overlap produced by two different heartbeats by a similarity index of the corresponding classification. A high similarity means that, even if the two heartbeats have a different shape, the week’s moments with the highest and lowest intensity of both types of crime are similar. In contrast, a low similarity means that one type of crime has a high intensity in the mornings and the other in the evenings, for instance. For $x = 20\%$ (as well as for $x=80\%$), any two heartbeats have an overlap of at least 60%, and two random classifications have an overlap in nearly 70% of the times. Therefore, if for two types of crime, the classification obtained for the top $x=20\%$ moments of the week is close to 70%, that similarity is no different than the one obtained by randomness. For different values of *x*, the similarity between any type of crime and robbery of a person shows that crimes follow substantially different temporal patterns (Fig. 6).

The overlap between the top $x\%$ moments of robbery of a person and other crimes shows a significant discrepancy. For example, the overlap of the top 10% moments of the week between the heartbeat of robbery of a person and of crimes suffered by people who deliver are close to being the ones obtained by randomness. For the most intense moments of the week, robbery of a person and carjacking have the highest discrepancy. Still, for the 40% least intense moments of the week (or less), theft and robbery of a person have the lowest similarity. The temporal trace of robbery of a person and theft looks more similar for the week’s moments in which crime is highly concentrated than the moments in which it is lowly concentrated.

There is also a high discrepancy of the heartbeats obtained for robbery of a person between different parts of the city, particularly for the week’s moments with the lowest intensity. The similarity between the classification in the city centre and any other region is similar to the one obtained by randomness. However, for the top 10% moments of the week, the agreement of the most intense moments of the week of all regions of the city is considerably high, marking the daily peaks observed with a high intensity of robbery of a person (Fig. 5).

Results show that the temporal concentration observed for different types of crime and distinct regions of the city, might be substantially different, even with different thresholds (see the “Seasonality and Shocks” of the Appendix).

### How Stable are the Heartbeats?

**Fig. 7:**
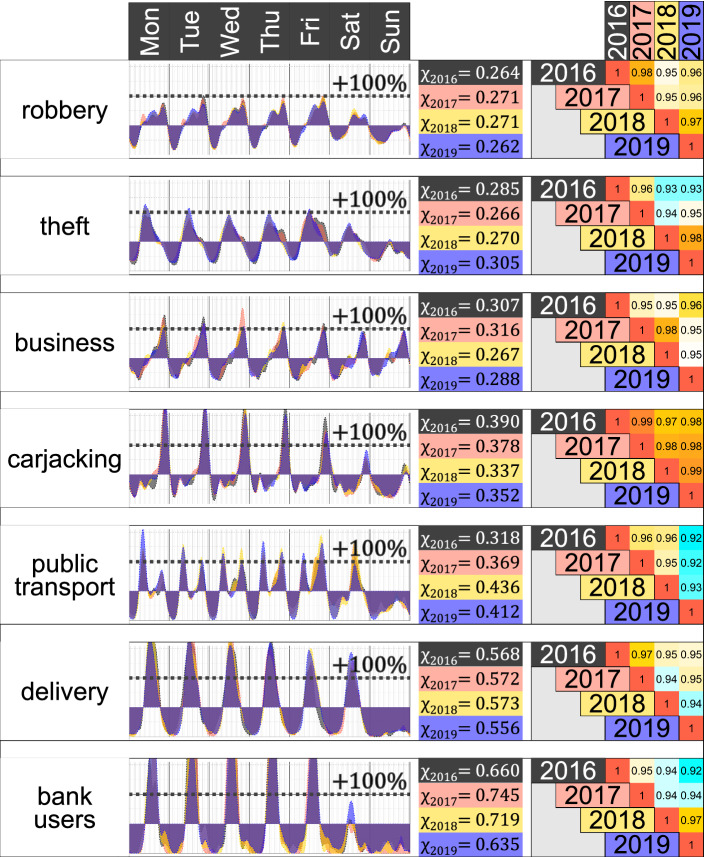
Heartbeat of each type of crime between 2016 and 2019 (left). Concentration metric for each year (middle) and agreement of the classification for the top 10% (right)

The observed heartbeats could result from the temporal aggregation of the data (considering more than 1500 days of crime) with a varying pattern through time. However, this is not the case. Constructing the heartbeats separately for crimes that occurred in 2016, 2017, 2018 and 2019 gives four full-year heartbeats for each type of crime, so it is possible to compare the correlation, the concentration, and the top moments of the week between distinct years. Results show that the yearly heartbeats for different types of crime are surprisingly stable (Fig. 7). In the case of robbery of a person, for instance, the concentration varies from $\chi _{R_{2019}} = 0.262$ to $\chi _{R_{2018}} = 0.271$, whereas their correlation ranges from 0.964 to 0.986. For other types of crime, the minimum correlation ranges from 0.962 for crimes suffered by people who deliver, to 0.862 in the case of theft.

The temporal concentration of crime varies slightly from one year to the next one. Statistically speaking, though, in some selected cases it is possible to reject that the heartbeat observed is the same across time since the concentration intervals do not intersect. There could be some perturbations from one year to the next on the temporal patterns of crime, which perhaps become even more visible for longer periods, or because of some significant shocks (such as the COVID-19 pandemic, for example). Still, although there is some variation between the heartbeats, the agreement between the top 10% moments of the week between distinct years is also very high. The level of agreement of the top 10% moments of the week is between 9.9 h (observed for crimes against public transport users) and 16.8 h (for carjacking). Yet, for a heartbeat computed for randomly distributed events in time, the agreement in the top 10% moments of the week gives $1.7 \pm 2.6$ h. See the “Similarity Between Years” of the Appendix for the metric of other temporal agreements.

Heartbeats, the weekly concentration of crime, and the week’s most crime-intense moments are very stable through the years. There is more variation in the shape and corresponding top moments of a heartbeat when different types of crime or regions are considered than the changes of the temporal trace of a specific type of crime through the years.

### Seasonality and Special Events

**Fig. 8:**
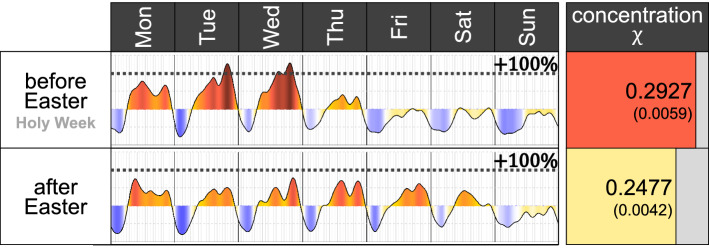
Weekly variation of the heartbeat of crime $\eta (t)$ and the concentration of crime $\chi$ obtained considering the robberies of a person which occurred the week before Easter each year (also called “Holy Week”) in the top part and the week after Easter in the bottom part. Each year Easter was celebrated on a different date: 27 March 2016, 16 April 2017, 1 April 2018 and 21 April 2019. There are more than 3200 reported crimes in the week before Easter and more than 4100 in the week after

The heartbeat is based on the intensity of crime through the week, and as such, it reflects the activities and routines of the area. Perhaps due to the high flow of people on a Metro station, students leaving their school, a dark alley or a bar that is open late at night, routines are quite stable and therefore, so are the heartbeats. In general, a heartbeat reflects the crime during a “regular week”. However, heartbeats might also change due to seasons or due to special events. Consider, for instance, the heartbeat of robberies of a person which occurred in Mexico City during the week before and the week after Easter. The week before Easter, also known as “Holy Week,” usually has less economic activities, schools are closed and the city has more leisure and religious activities. In a country with many Catholic traditions, Thursday to Sunday (Easter day) are usually bank holidays. And, the heartbeat reflects the decrease in activities during the Holy Week (Fig. 8). The hottest Wednesday of the year, in terms of robberies, happens during Holy Week, and a couple of days later, the coldest Friday of the year occurs on Good Friday.

Although in many countries, the Monday after Easter is also a bank holiday, this is not the case in Mexico. Therefore, the week after Easter, things almost go back to normal, with hotter Thursdays and Fridays. Other bank holidays and special events also affect the heartbeat and likely, the surrounding days as well. The heartbeat of crimes in the city during the Day of the Dead in Mexico City (2 November), or the day that Mexican men’s national football team plays against another team, for instance, will not have the “regular” heartbeat, because the day is not regular either. The season, or whether it is a bank holiday or Christmas, also changes the heartbeat of the crime signal (see the “Seasonality and Shocks” of the Appendix for more details on the seasonality and other special events).

## Conclusions and Discussion

Crime is highly concentrated in a few places (Weisburd 2015), mostly suffered and committed by some individuals (SooHyun et al. 2017; Martinez et al. 2017), and also, here it is observed that crime is highly concentrated in the moments of the week in which it happens. A new function to detect and visualise the weekly patterns of crime, its variation around the mean and a new metric for the concentration of crime was constructed here. The new function, the heartbeat of the crime signal, does not depend on arbitrary grids, counts of crimes every 4 or 6 h and overcomes the issue that most crimes are reported at even hours. Furthermore, the heartbeat allows classifying distinct moments of the week as having a ‘high’ or ‘low’ intensity, and such classification can be compared between different types of crime and regions.

Using data corresponding to violent crimes reported to the police in Mexico City, it was detected that crime is highly concentrated during different week moments. Yet, the heartbeat is not a fixed curve, but varies progressively through the years, is not the same for different types of crime, and changes across distinct neighbourhoods. For example, during the top 10% moments of the week (which corresponds to 16.8 h on a week or 2 h and 24 min each day), 44% of the robberies of a bank user happen, but only 17% of the robberies of a person. In the case of robbery of a person, the top 10% moments of the week of the city centre concentrate 23% of the intensity, but in the three neighbouring concentric areas, the top 10% moments of the week concentrate a smaller percentage.

Some caution is needed to construct the heartbeat of temporal events. With a reduced number of events (so a limited amount of data), a heartbeat will be obtained, and some spurious pattern might be observed, particularly with a very reduced number of events. The same happens on a hot spot map with only a few crimes, frequently showing some spurious concentrations. However, this could be the result of randomness combined with a limited amount of data. Under such circumstances, it is worth asking if the heartbeat changes drastically if one or a few events are missing, in which case it might be difficult to conclude many things from the data. In addition, it is relevant to check if the observed temporal signature of the events reflects any concentration, if that level of concentration could be observed simply by randomness and if that concentration is somehow stable (Hipp and Kim 2017).

The heartbeats allow to compare the correlation of the weekly intensity of distinct types of crime and detect a considerably low correlation for some types of crime, for instance, carjacking and crimes suffered by bank users. As a result of the weekly concentration of crime, it is observed that the intensity of crime varies considerably, with moments of the week with nearly no crimes (particularly for certain types of crime) and moments with a peak intensity. For example, for robberies of a bank user, the peak intensity, observed from Wednesday to Friday at around noon, can reach up to $+\,400\%$ the average intensity, but it is nearly zero on Sundays.

The temporal concentration of crime, particularly in the case of robberies with a present victim, reflects the routines and activities of different neighbourhoods of the city. Except maybe for special events or holidays, the heartbeat is quite stable, meaning that we can, to a certain extent, know when a crime is more likely to occur. Still, there are open questions with respect to the heartbeat of the crime signal, for example, whether rainy days have a significantly different signature than other days, whether a similar heartbeat of robberies is observed, for instance, in Cape Town, Rio de Janeiro or Manila or whether the presence or absence of police significantly alters the shape.

It has been shown that a place-based policing and a tactical police response can lead to significant crime reductions without displacement (Santos and Santos 2015) which last for some weeks in those urban and suburban neighbourhoods (Santos and Santos 2021; Gill et al. 2017). Therefore, heartbeats could also be combined with hot spot maps to detect locations prone to suffering crimes, designing and improving police practices for resources allocation and reducing response time to emergency calls. Furthermore, adding heartbeats to the standard hot spot maps provides easy visualisation of crime’s spatial and temporal patterns (Townsley 2008).

## Figures and Tables

**Fig. 9:**
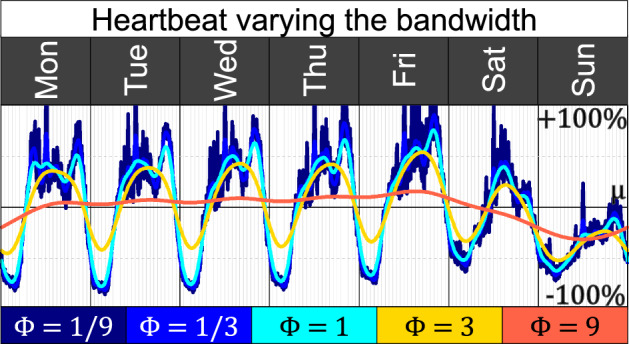
Heartbeat of all property crimes reported between January 2016 and March 2020 in Mexico City, varying the bandwidth. The value of the bandwidth used for constructing the heartbeats is $\phi \omega$, where $\omega$ is the original value. Thus, with $\phi = 1$, the original heartbeat is observed. With $\phi = 3$, a bandwidth three times wider is used. With $\phi = 9$, the heartbeat is nearly flat and has a little variation on a single day. In contrast, with $\phi = 1/9$, the heartbeat oscillates rapidly, so fast that on a Friday, for example, the heartbeat goes from values close to 10% to values above 100% and back to 10% in less than 10 min

**Fig. 10:**
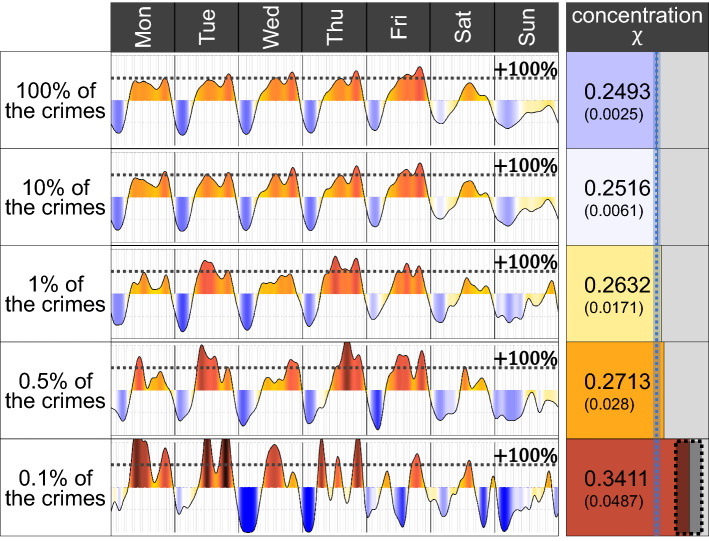
Weekly variation of the heartbeat of crime $\eta (t)$ and the concentration of crime $\chi$ obtained considering all the crimes between January 2016 and March 2020 (top part). Sampling 10% of the crimes, or 1%, 0.5% and 0.1% different heartbeats and levels of concentration are observed. With a sample size of 10%, the heartbeat is constructed with more than 22,000 crimes, but with a sample size of 0.1%, only 223 crimes are used. With a sample size of 0.1% of the crimes, an interval for the concentration metric $\chi$ does not overlap the observed concentration with no sampling

**Fig. 11:**
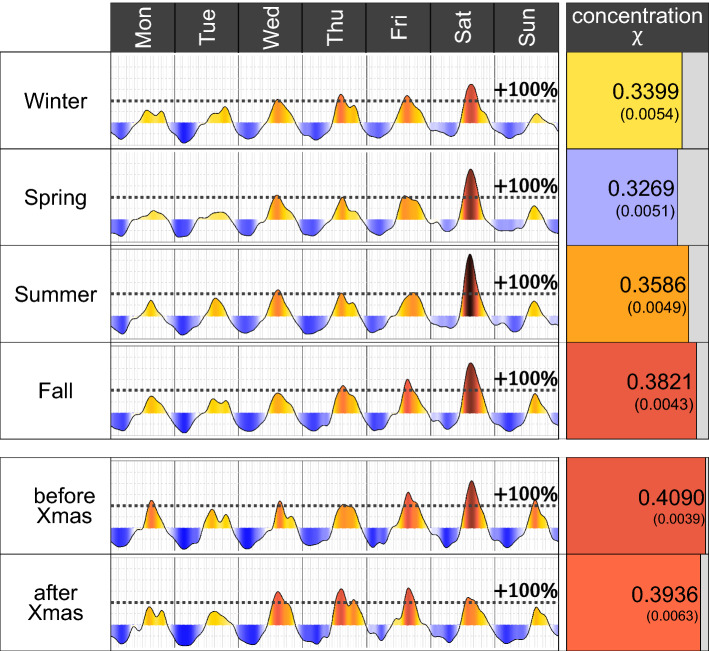
Heartbeat of robberies of a person across different seasons (top) and before and after Christmas (bottom)

**Fig. 12:**
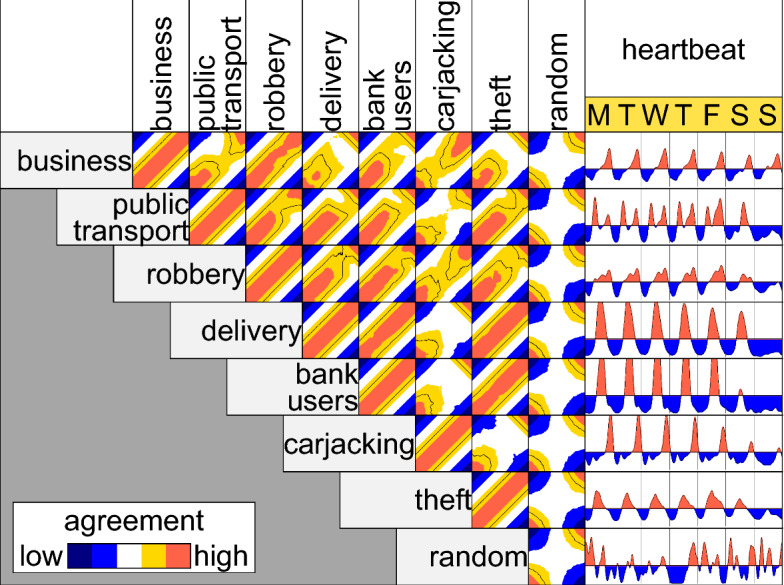
Level of agreement of the binary classification between the top and the bottom moments of the week. For a pair of crime types, the top $x$ moments (horizontal) and the top $y$ moments (vertical) give an agreement, marked by different colours. The diagonal compares one type of crime against itself, where the only variation comes from the different values of $x$ and $y$. Types of crime for which the classification is more similar look like the observed colours in the diagonal, such as robberies of a bank user and crimes against people who deliver. A randomly constructed heartbeat is also considered, which gives the least agreement between classifications

**Fig. 13:**
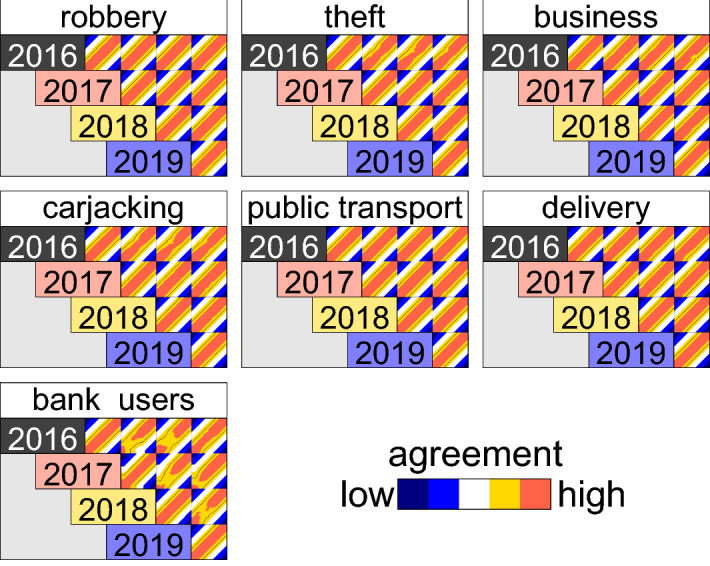
Agreement of the binary classification of the week’s moments for different years, considering each type of crime separately. For each pair of years, the top $x\%$ moments (horizontal) and $y\%$ moments (vertical) is coloured according to the agreement of the corresponding binary classification. The diagonal is the classification obtained comparing one year against itself. The highest variation of the binary classification is observed for robberies of a bank user comparing the 2016 and 2018 heartbeats

## Appendix

### Mathematical Formality

#### A Scaled Kernel

Formally speaking, the function

3 $$\begin{aligned} G(t) = \kappa \exp \left( -\frac{(t-s_i)^2}{2\omega ^2} \right) , \hbox { with}\ t \in [0, 7) \end{aligned}$$

is a distribution, for a fixed value of $s_i \in [0,7)$, for some bandwidth $\omega >0$ and for a value of $\kappa = 1/\int _0^7 \exp \left( -\frac{(x-s_i)^2}{2\omega ^2} \right) \, dx$ such that $\int _0^7 G(t) \, dt = 1$. The impact of $\kappa$ is to scale the function *G*(*t*) to a certain height so that the area under the curve in the [0, 7) interval is equal to one. The value of $\kappa$ varies depending on the bandwidth, $\omega$ and there is no analytical expression to compute its value. However, the effect that $\kappa$ has on the function *G*(*t*) is only to scale it. Since the mean ${\bar{G}}$ is also scaled, once the weekly variation is considered, the function $\gamma (t) = (G(t) - {\bar{G}})/{\bar{G}}$ no longer depends on the value of $\kappa$. Thus, $\gamma (t)$ is scale invariant. Also, the Gini index is scale invariant, meaning that the index of any vector remains the same if the vector is multiplied by a positive constant. Therefore, the expression for computing the heartbeat (Eq. 1) could also contain some constant $\kappa >0$ since the function *H*(*t*) is, fundamentally, the sum of many functions that look like *G*(*t*). However, the weekly variation of the heartbeat $\eta (t)$ and the concentration metric $\chi$ remain the same with any positive value of $\kappa$, and there is no real gain (quantitative or in terms of interpretation) on using any value of $\kappa$. Therefore, the heartbeat *H*(*t*) can simply be considered a scaled version of the distribution and, as a rule favouring a simple expression, take a value of $\kappa = 1$. This helps us ignore the constant in Eq. 3 and obtain a simple version in Eq. 1.

#### A Discrete Gini Index

The Gini index has been used to measure crime concentration (Prieto Curiel and Bishop 2016; Bernasco and Steenbeek 2017; Chalfin et al. 2021) and it is most commonly defined for discrete observations (Dorfman 1979). The heartbeat is a continuous function for which the Gini index can also be defined. Let $F(y) = \theta \int _0^y H(t) \, dt$ be the cumulative distribution function of the some heartbeat *H*(*t*), where the parameter $\theta$ is such that $\theta \int _0^7 H(t) \, dt = 1$. An expression for the Gini index (the concentration metric $\chi$) is given by

4 $$\begin{aligned} \chi = 1 - \frac{1}{\mu } \int _0^7 (1 - F(y))^2 \, dy, \end{aligned}$$

where $\mu$ is the mean value of the heartbeat (Dorfman 1979). The expression of the concentration metric depends on the data considered and does not have an analytical solution. A more straightforward technique is to consider a discrete version of the heartbeat. Formally, divide the domain of the heartbeat (the [0, 7) interval) into *n* segments, $0 = x_1, x_2, \dots , x_n \approx 7$, consider the value of the heartbeat for each segment, $H(x_1), H(x_2), \dots , H(x_n)$ and consider the (discrete) Gini index of the corresponding *n* values of the function by sorting the values of $H(x_k)$ in an increasing order and computing the Lorenz curve. With enough segments (which depends on how smooth the function is), the *n* values of the function can be used to compute a discrete version of the Gini index. In the case of a heartbeat, the domain of *H*(*t*) is the [0, 7) interval. With $n > 1000$, a week is divided into intervals of less than 10 min each, and the (discrete) Gini index gives practically the same result with an even more refined partition. Therefore, the concentration metric $\chi$ is computed considering the discrete expression of the heartbeat by applying the formula for the discrete Gini index by dividing the [0, 7) interval into $n = 1000$ segments.

#### Altering the Bandwidth of a Heartbeat

Different values of the bandwidth $\omega$ alter the heartbeats and the concentration. With a smaller bandwidth, a coarse and rugged heartbeat is obtained, whereas with larger bandwidths, a smoother heartbeat is obtained (Fig. 9). A smooth heartbeat might miss some valuable details on the daily patterns, whereas some apparent concentrations might appear with a small bandwidth, particularly if the data is not very precise. In general, observing the impact of $\omega$ on a heartbeat is recommended, and different values should be analysed. For crime reported to the police, a fixed value of the bandwidth was picked, independent of the number of crimes considered. Other options also exist, for example, where the additional precision gets translated in more confidence and a narrower smoothing process with more data. Yet, in the case of reported crime, there is a high tendency to write them at even hours or 30-min intervals, so having a fixed value of the bandwidth prevents the heartbeat from gaining too much confidence based on data that lack such confidence.

The heartbeat, with a fixed value of the bandwidth, allows comparing very different volumes of crime and observe similar results. For example, the heartbeat of robberies of a bank user (1883 crimes) and robberies of a person (96,711 crimes) was compared (Fig. 3) even if the second type of crime is more than 50 times more intense than the first one.

### Considering only Property Crimes with a Present Victim

Only property crimes where the victim is present were considered to construct the heartbeats. Other types of crime have issues to analyse the moment in which they happened. The frequency of different types of crime reported to the Police in Mexico City between January 2016 and March 2021 (Table 2) shows that the most frequent type of crime in the city is theft where the victim notices their missing property after the crime, such as a wallet or a mobile phone being stolen, so the time of the crime is not clear. The moment in which domestic violence is reported might refer to many aspects related to that type of crime, and so the heartbeat of domestic violence or of property crimes where the victim is not present is not easy to interpret. Non-intentional traffic events (including injuries and non-intentional homicide) also show a temporal pattern (Prieto Curiel et al. 2021), but it is not an intentional event, and so they should be considered separately. Severe crimes (including intentional homicide, gunshots, rape, kidnap and missing person) are very scarce, and the time and location of the report does not always correspond to the event (for example, more than 40% of the gunshots are registered in a hospital). Finally, there are roughly 46.8 crime of lesser seriousness each day, such as vandalism, partial car theft, threats, gang fights and many more.

**Table 2:** Daily frequency of some types of crimes reported to the Police in Mexico City between January 2016 and March 2020

Type of crime	Daily rate	%
Crimes without a present victim	339.9	51.6
Property crimes with a present victim	143.7	21.8
Domestic violence	86.6	13.1
Traffic events	29.6	4.5
Severe crimes	12.5	1.9
Others	46.8	7.1
Total	339.9	100

Roughly 143.7 property crimes where the victim is present occurred each day. For other types of crime, either because of their low frequency, lack of precision, or difficulty interpreting its meaning, the corresponding heartbeats are not considered. The intensity of property crimes where a victim is present varies from an average of 90.3 crimes each Sunday to an average of 166.2 crimes each Friday (Table 3).

**Table 3:** Daily rate of property crimes with a present victim according to the day of the week

Day	Mon	Tue	Wed	Thu	Fri	Sat	Sun
Rate	154.5	152.2	154.1	155.2	166.2	133.4	90.3

### Under-Reported Crime

Crimes are not always reported to the police and considering only reported crimes could bias the perceived temporal patterns. In Mexico City, less than 6% of the crimes are reported to the police, although it is not uniform. For instance, more than 60% of the car thefts but less than 3% of the extortions are reported to the police (INEGI 2019). Thus, it is relevant to consider that frequently the data available is only a small part of a bigger reality.

Having less than 6% of the crimes to construct the heartbeats could significantly alter the results for two reasons. Firstly, if the data is biased, for example, if crimes which happened during the night, or the weekend, are less likely to be reported to the police. However, reporting a crime in Mexico City requires the victim to go to a police station (a *Ministerio Público*) to provide a detailed description of the event, and the process usually takes some hours. Thus, people tend to report their crimes many hours (perhaps even days) after suffering them. With no other data source available for validation or correction, this type of bias cannot be easily controlled and has to be ignored.

The second type of bias that could result from such a low frequency of reported crimes comes from having a small percentage of the data. Potentially, a minimal dataset restricts the possible interpretation of the results. This restriction happens, for instance, on some election poll based only on a dozen surveys. Even if a limited number of surveys are an unbiased representation of reality, little can be inferred from them since most perceived patterns could result only from randomness.

However, having only 6% of the data, as in the case of Mexico City, is sufficient for constructing the heartbeat. Between January 2016 and March 2020, more than 222,000 robberies with a present victim were reported, meaning that more than 3.7 million robberies were suffered in those 51 months. If the available data is considered a random sample of the whole robberies (random sample only with respect to the time in which crimes were suffered) then, it is possible to filter even more the data and detect the impact of such thinning process. In other words, the heartbeat might be constructed by resampling the observed data and obtain intervals as one of the results, but also, it is possible to sample only 10% of the data and construct the corresponding heartbeat, or with 1% of the data or even 0.1% of the data (Fig. 10). With only 1% of the data, the heartbeat is constructed using only 2200 crimes, and still, its shape (with colder nights and weekends, with peaks in the evening) is similar to the one constructed with 100% of the data. Also, the concentration metric $\chi _{1\%}$ and its interval overlap with the metric for the whole robberies, meaning that we would not reject a null hypothesis that they are constructed from the same distribution. There is a limit to how thin the data can be. With 0.1% of the crimes (only 223 crimes) the resulting heartbeat no longer captures the same temporal signature. The interval for the concentration metric $\chi _{0.1\%}$ does not overlap with the metric for the whole robberies, meaning that a null hypothesis that both heartbeats are obtained with data that comes from the same distribution would be rejected.

Although only 6% of the crimes are reported in Mexico City, they still provide thousands of dates to construct heartbeats. Even if fewer crimes (obtained randomly) were reported to the police, it would still be enough information to build almost the same heartbeat. The same can be considered if more crimes were reported to the police. If instead of 6% of the victims, 18% or even 54% would report their crimes, it would not alter much the heartbeat or the intervals.

The volume of data, however, is crucial. With roughly 1000 crimes, it is still possible to obtain some precision on its temporal pattern, but with fewer crimes, the level of certainty is lost, and the intervals become too wide. In turn, it means that obtaining, for instance, the heartbeat or robberies of a bank user (1883 crimes in total) by year (roughly 440 reported crimes per year) or near the city centre (101 crimes at a distance smaller than 2.5 km from the city centre) might not give a precise idea of the temporal distribution of the crimes because not enough victims report them. If instead of 6% of the crimes, 60% of the crimes were reported to the police, the heartbeat of robberies of a bank user by year and by region could be constructed and compared with more confidence in the results. The fact that such a small percentage of crimes are reported to the police does limit some of the results and the possible applications of the heartbeats.

### Seasonality and Shocks

Everyday life, characterised by daily and weekly routines, explains why crime occurs in recurrent spaces and times, but these routines might change according to the season or other aspects, such as holidays or festivities. Considering the robberies of a person inside a disc centred in the main square with a 2.5 km radius, it is possible to compare the heartbeats during different seasons of the year (Fig. 11). During Winter (January–March), the peaks observed on Saturday and Sunday, for instance, are not as high as they are during the other seasons, particularly during Summer. Some of the seasonal differences might be the result of different weather conditions or activities that vary yearly. For example, during the Summer, the city centre goes below the average between 19:00 and 20:00, but during other seasons the city is going through a peak at those times of the week. The lowest concentration $\chi _S = 0.3269$ is observed during the Spring and has the highest concentration during the Fall, with $\chi _F = 0.3821$. They are significantly different, with intervals obtained by sampling from the same set of robberies.

Also, although not seasonal, some differences in the heartbeat of robberies happened the three weeks before or the three weeks after Christmas (Fig. 11, bottom part). For example, before Christmas, there are more activities in the city centre, and from Friday to Sunday, there are higher peaks and activities that finish later during the day. After Christmas, the weekend peaks are not as high. Other seasonal patterns or differences due to events might also be observed.

### Similarity Between Distinct Heartbeats

The similarity observed for the binary classification of two types of crime for different thresholds *x* and *y*, that is $I_{\eta _c}(t, x)$ and $I_{\eta _b}(t, y)$, shows that, in general, distinct types of crime have different moments of the week in with a high and low intensity (Fig. 12). Further, the weekly concentration pattern observed between carjacking and robberies of a bank user is so different that they display a similarity close to what would be observed by randomness.

### Similarity Between Years

The agreement of the top $x\%$ and the top $y\%$ moments between two years show, in general, a very high agreement both for the most and the least intense moments of the week (Fig. 13).

For example, in the case of robbery of a person, the 2016 and the 2019 heartbeats classify the top and bottom moments of the week with almost a perfect agreement. Some disagreement is observed for robberies of a bank user, where between 2017 and 2018, for example, there is some mild disagreement in the classification obtained for the top 30% moments of the week. There is a low frequency of robberies of a bank user, with 0.94 crimes per day in 2017 and 1.04 in 2018, compared to the 85.3 robberies of a person reported each day (on average) in 2018. The low frequency of the data does have an impact on the precision and stability of the heartbeats. Considering the binary classification produced by each heartbeat, the highest agreement is observed for carjacking (0.97–0.99 between different years), meaning a more stable pattern between distinct years, and the lowest is for crimes suffered by public transport users (0.92–0.96).
